# Engineering bacteriophages for enhanced host range and efficacy: insights from bacteriophage-bacteria interactions

**DOI:** 10.3389/fmicb.2023.1172635

**Published:** 2023-05-31

**Authors:** Huang-Jie Jia, Pan-Pan Jia, Supei Yin, Ling-Kang Bu, Guan Yang, De-Sheng Pei

**Affiliations:** ^1^College of Resources and Environment, University of Chinese Academy of Sciences, Beijing, China; ^2^Chongqing Institute of Green and Intelligent Technology, Chinese Academy of Sciences, Chongqing, China; ^3^School of Public Health, Chongqing Medical University, Chongqing, China; ^4^Urinary Nephropathy Center, The Second Affiliated Hospital of Chongqing Medical University, Chongqing, China; ^5^College of Life Science, Henan Normal University, Xinxiang, China

**Keywords:** multidrug resistant bacteria, phage engineering, gene editing, phage-host interaction, high-throughput methods

## Abstract

Bacteriophages, the most abundant organisms on earth, have the potential to address the rise of multidrug-resistant bacteria resulting from the overuse of antibiotics. However, their high specificity and limited host range can hinder their effectiveness. Phage engineering, through the use of gene editing techniques, offers a means to enhance the host range of bacteria, improve phage efficacy, and facilitate efficient cell-free production of phage drugs. To engineer phages effectively, it is necessary to understand the interaction between phages and host bacteria. Understanding the interaction between the receptor recognition protein of bacteriophages and host receptors can serve as a valuable guide for modifying or replacing these proteins, thereby altering the receptor range of the bacteriophage. Research and development focused on the CRISPR-Cas bacterial immune system against bacteriophage nucleic acids can provide the necessary tools to promote recombination and counter-selection in engineered bacteriophage programs. Additionally, studying the transcription and assembly functions of bacteriophages in host bacteria can facilitate the engineered assembly of bacteriophage genomes in non-host environments. This review highlights a comprehensive summary of phage engineering methods, including in-host and out-of-host engineering, and the use of high-throughput methods to understand their role. The main aim of these techniques is to harness the intricate interactions between bacteriophages and hosts to inform and guide the engineering of bacteriophages, particularly in the context of studying and manipulating the host range of bacteriophages. By employing advanced high-throughput methods to identify specific bacteriophage receptor recognition genes, and subsequently introducing modifications or performing gene swapping through in-host recombination or out-of-host synthesis, it becomes possible to strategically alter the host range of bacteriophages. This capability holds immense significance for leveraging bacteriophages as a promising therapeutic approach against antibiotic-resistant bacteria.

## 1. Introduction

Phages, viruses that infect bacteria, are the most abundant and ubiquitous organisms on the planet (Balcazar, 2014; Islam et al., 2019), with an estimated number of 10^31^ phages worldwide (Czajkowski, 2019). The study of phages and their interactions with their bacterial hosts has been a driving force behind the development of molecular biology, from the initial experiments demonstrating that DNA is the genetic material of living cells, using phages as a model system (Hershey and Chase, 1952), to the discovery and application of temperate bacteriophage-based genetic integration tools, such as the lambda red recombinases (Fehér et al., 2012), and the development of the gene-editing system RM/CRISPR (Marraffini, 2015). Phages have now become a crucial model organism for understanding all aspects of modern molecular biology (Subedi and Barr, 2021).

The extensive usage of antibiotics globally has led to the emergence of multidrug-resistant (MDR) strains in many pathogenic bacteria (Sun et al., 2022; Ul-Hamid et al., 2022). Phage therapy, which involves the use of phages that specifically infect and kill the host bacteria without affecting other bacteria, is a promising strategy against MDR (Rodriguez-Gonzalez et al., 2020; Kim et al., 2021; Salazar et al., 2021; Jia et al., 2023). However, the high host specificity and narrow host range of most phages limit their effectiveness for use in phage therapy (Zhan et al., 2015; Jeon et al., 2016). The emergence of these limitations can be attributed primarily to two factors. Firstly, host bacteria can evade infection by narrow-host-range phages through mechanisms such as evolution or immunity. This evasive behavior leads to the development of phage resistance in the target pathogenic bacteria (Pires et al., 2017; Peng and Chen, 2021). Secondly, there exist significant variations among clinical isolates of bacterial pathogens within the same species. Consequently, if a phage is unable to infect and eliminate both bacterial strains, a phage that proves effective for one patient may not be suitable for another patient (Hatfull et al., 2022). The use of a phage cocktail containing multiple phages that infect the host bacteria through different mechanisms has proven to be an effective solution (Esteves et al., 2021; Zhang et al., 2021). Moreover, employing a combination of phages that target distinct receptors on the surface of the same host bacterium can effectively impede the emergence of phage resistance within the bacterial population. For example, when clinicians utilize a cocktail preparation consisting of two phages, it is crucial for these phages to possess minimal or no shared genes. This ensures that they are unlikely to rely on the same bacterial receptor, thereby reducing the risk of mutations in a single receptor that could confer resistance against both phages (Mutalik et al., 2020; Hatfull et al., 2022). The process of screening for phages with diverse invasion pathways from nature is a demanding and time-consuming task. However, an alternative approach to broaden the host range of phages and enhance their effectiveness in phage therapy is through phage engineering using gene editing techniques. Cell-free phage production can also facilitate the efficient production of phage drugs. To effectively modify phages for therapy, it is essential to have a comprehensive understanding of the mechanism underlying the interaction between phages and their host bacteria.

Previous reviews have systematically described the complex and diverse process of the interaction between bacteriophages and host bacteria (Hampton et al., 2020). Bacteria have evolved various antiviral defense systems against infection by their viruses and mobile genetic elements, including nine identified anti-phage systems that can provide specific or broad defense, such as RM, CRISPR, and Retron (Payne et al., 2022). Besides, bacteria can prevent bacteriophage infection by reducing attachment through mutations in surface bacteriophage receptors (Gurney et al., 2019). Under the defensive pressure of the host bacteria, bacteriophages continually evolve, with mutations allowing their DNA to evade systemic targeting defense system cleavage and mutations in their receptor-binding proteins enabling them to attach to mutated receptors on the host surface (Lee et al., 2016; Yuan et al., 2019; Broniewski et al., 2020). Furthermore, bacteriophage populations can exchange receptor-binding protein genes, leading to significant changes in their host range to adapt to environmental pressures (Xiao et al., 2018; Górniak et al., 2022). The discovery of these mechanisms provides insights into the application of gene editing systems, which have been widely used in biological organisms. Bacteriophages, as the origin of these tools, have been naturally utilized in engineering modifications.

This review presents an overview of the various methods for bacteriophage engineering, including both in-host and ex-host gene editing techniques. We focus on the strategies for modifying the host range of bacteriophages, specifically the alteration of their receptor-binding proteins. These engineering methods are all based on the understanding of the mechanisms underlying the interaction between bacteriophages and their host bacteria. Besides, we discuss high-throughput methods for studying this interaction, which can facilitate the development of more effective bacteriophage engineering approaches. In summary, the phage-bacteria interaction not only provides us with valuable tools for phage engineering but also offers guidance for the further improvement of these techniques.

## 2. Phage gene editing in host bacteria

### 2.1. Gene editing of phages using recombination

**Figure 1:**
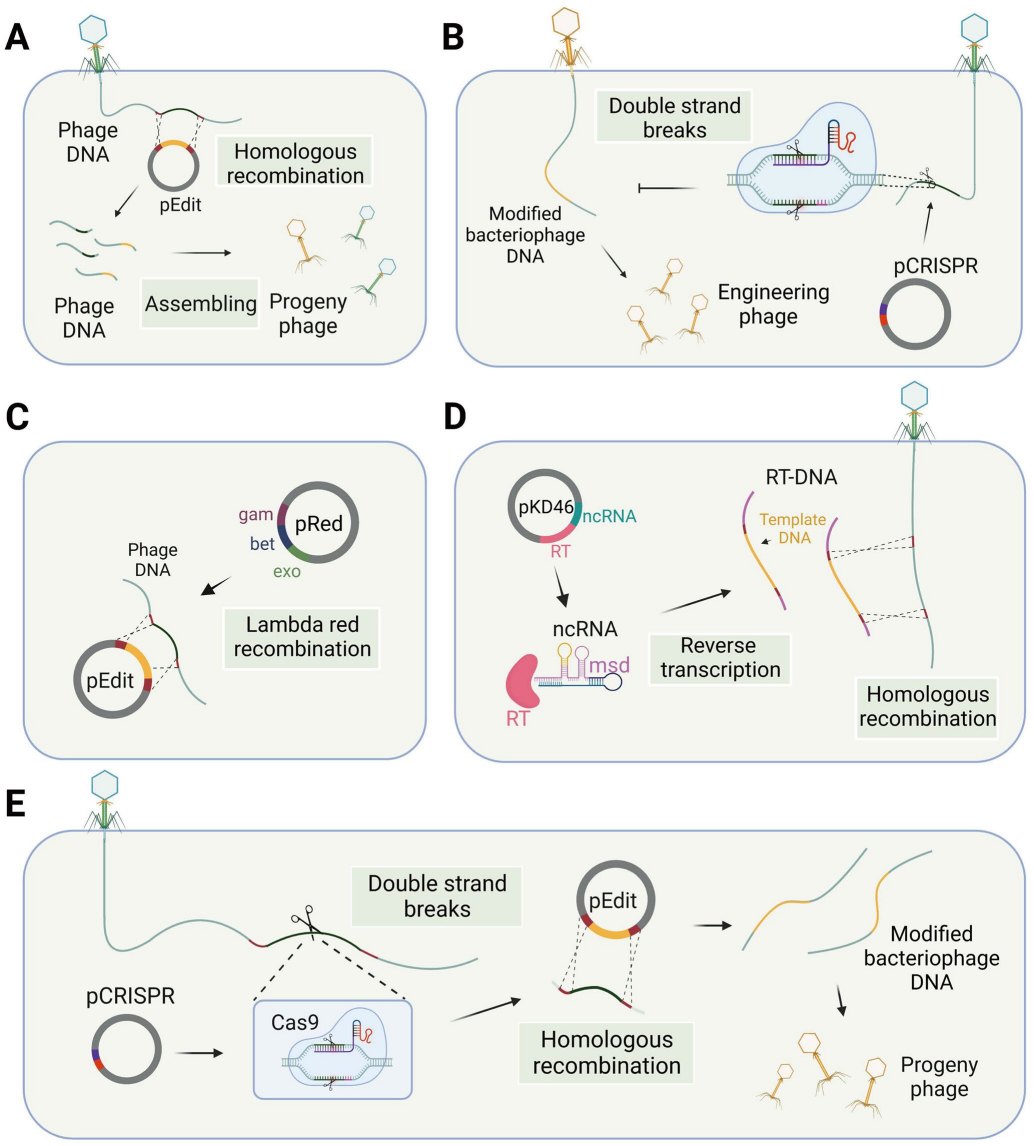
Phage gene editing technology in the host bacteria. **(A)** Phage genome editing through homologous recombination. **(B)** Differentiation of wild-type and engineered phages using the CRISPR-Cas system. **(C)** The lambda red recombination system facilitates recombination between editing templates and phage DNA in bacterial cells. **(D)** Modified retrons generate editing templates via reverse transcription in the host bacterium to facilitate homologous recombination. **(E)** CRISPR-Cas system utilized for modifying phage genes in host bacteria (single-plasmid-mediated asynchronous recombination, images are created with Biorender.com).

The genome of a temperate bacteriophage can integrate into the host chromosome, forming a prophage. This integration allows for stable incorporation and makes the bacteriophage genome amenable to nucleic acid editing using the same methods employed for bacterial genomes. Besides, gene editing of virulent bacteriophages necessitates specialized methods. Traditional methods for modifying lytic phage genes involve the use of homologous recombination technology (Court et al., 2002), which takes place within the host bacteria. In this process, a DNA template sequence with homologous arms is transfected into the host cells, and the bacteriophage DNA is edited during subsequent infection of the host bacteria (Figure 1A). The edited bacteriophage DNA, packaged in a protein coat, forms the engineered bacteriophage progeny. However, it is worth noting that the generation of recombinant phages through this method results in only a small fraction of the progeny phages. The reported rates of recombination vary between 10^−10^ and 10^−4^ (Pires et al., 2016), primarily due to the inherent challenges posed by low recombination and transformation efficiency associated with this technique.

For gene editing of lytic bacteriophages, recombination needs to occur during the process of phage infection of the host. Shorter recombination times are a significant factor leading to low recombination efficiency. Researchers have made efforts to enhance the activity of the recombination enzyme to increase recombination efficiency. Some phage genomes contain their own recombinase enzymes, which exhibit superior performance in bacterial hosts compared to the RecA-mediated pathway and also possess better tolerance for mismatches (Manning and Dokland, 2020), although this process may result in the formation of recognition sequence scars. Consequently, in the case of this particular type of phage, the presence of a homologous template within the infected bacterial cell is adequate to generate recombinant phage progeny (Muniyappa and Radding, 1986). However, gene modification efficiency by recombination in some phages is not optimal, which may be due to the absence of self-contained recombinases in these phages. The lambda red recombinases, sourced from bacteriophages and comprising three proteins Exo, Beta, and Gam, can efficiently achieve recombination of exogenous fragments through the cooperation of these three proteins (Figure 1C). Therefore, the simultaneous introduction of the lambda red recombinase system and template DNA into the host bacterium during the editing process of *Escherichia coli* phages can facilitate recombination and enhance editing efficiency (Oppenheim et al., 2004; Marinelli et al., 2008; Thomason et al., 2009; Fehér et al., 2012).

The low transformation rate of host bacteria is a significant limiting factor when it comes to editing the phage genome within the host cell. This difficulty in importing template plasmids and exogenous recombination systems into the host cell hinders the improvement of recombination efficiency. Efficient transformation protocols have not been widely developed for many bacteria, particularly gram-positive bacteria with thicker cell walls. To overcome this issue, bacteriophage electroporation-based DNA recombination (BRED) has emerged as a highly efficient strategy. BRED employs electroporation to facilitate the transfer of phage DNA, template DNA, and exogenous recombination systems, such as the lambda red system or RecE/RecT-like proteins (Marinelli et al., 2008), into bacterial cells. A study conducted by Marinelli et al. (2008) demonstrated that this approach significantly enhances the level of phage gene recombination in *Mycobacterium smegmatis*, a gram-positive bacterium. The BRED technique achieves a high level of recombination efficiency by introducing suitable exogenous recombination systems into bacterial cells. However, it is essential for plasmid-based exogenous recombination systems to be efficiently transformed into cells and function effectively within them.

Efficient methods for selecting modified phages are essential due to the abundance of unmodified progeny phages produced in bacteriophage genetic engineering. For temperate phages, antibiotic resistance-genes can serve as markers, and conventional microbiological methods can be employed for screening. When the antibiotic resistance gene is recombined into the prophage, it imparts antibiotic resistance to the host bacteria, allowing for straightforward identification. Besides, specialized screening methods are necessary for virulent phages. One such method involves introducing marker genes, such as beta-galactosidase (Revel et al., 1961) or fluorescence protein (Yoichi et al., 2005), to facilitate plaque screening. The approach mentioned above still leads to the production of a considerable number of non-target progeny. However, an alternative method exists that can substantially decrease the occurrence of these non-target progeny, thus demonstrating a remarkable level of screening efficiency. This method leverages the utilization of genes from the bacterial host that are essential for phage invasion and replication but not crucial for host survival. For example, it has been established that the thioredoxin encoded by trxA and the dCMP kinase encoded by cmk gene are crucial during T7 bacteriophage infection of *Escherichia coli* (Qimron et al., 2006; Grigonyte et al., 2020). However, these genes are not essential for *Escherichia coli*’s growth. By inserting such genes into the template DNA and screening with host bacteria lacking these genes, one can select marked mutant bacteriophages (Qimron et al., 2006; Yosef et al., 2017). This method requires gene knockout of the host bacteria, and it can be challenging to identify appropriate screening genes for unknown bacteria and bacteriophages. Nevertheless, high-throughput methods like transposon sequencing and iCRISPR technology can study the genes involved in bacteriophage invasion in the host bacteria, and suitable screening genes can be selected from these research libraries. Both approaches for screening modified progeny demonstrate high efficiency, with the introduction of marker genes being the more convenient method. However, even with this method, a substantial number of unmodified phage progeny is still observed. On the other hand, the introduction of host genes requires modification of the host bacteria but eliminates the production of unmodified progeny. Additionally, an increasing number of studies have reported the utilization of the CRISPR-Cas system for counter-selecting modified progeny bacteriophages.

### 2.2. Gene editing of phages using CRISPR-Cas system

CRISPR-Cas was initially discovered as a component of the bacterial immune system and plays a crucial role in phage resistance (Barrangou et al., 2014; Lander et al., 2015; Fu et al., 2017). The mechanism has been extensively documented in various reports (Barrangou et al., 2014; Lander et al., 2015). The CRISPR-Cas system, which is based on adaptive immunity, RNA-guided DNA cleavage, and target specificity, has been developed into a powerful gene editing tool (Jinek et al., 2015a). Its gene editing potential has become more apparent over time, driving increased research and development in the field.

The CRISPR-Cas system is derived from the immune response of host bacteria against phages, making it an appropriate tool for engineering phages within bacterial cells. There are two main applications of the CRISPR-Cas system, specifically targeting the DNA strand, in the gene editing of bacteriophages. The first application involves introducing the CRISPR-Cas system into host bacteria, which are then modified to be resistant to wild-type bacteriophages rather than the modified genotype. After the recombination step, counter-selection is performed to identify the modified bacteriophages among the progeny. The second application involves co-transforming the CRISPR-Cas system and template DNA into host bacteria. The CRISPR-Cas system induces double-strand breaks at the target site, thereby enhancing recombination at the desired locus. Consequently, two gene editing strategies have been developed: single-plasmid-mediated asynchronous recombination (SPMAR) and dual-plasmid-mediated synchronous recombination (DPMSR) (Zhang X. et al., 2022). In the SPMAR approach, recombination (Figure 1A) and counter-selection (Figure 1B) are performed sequentially in different bacterial cells, while in DPMSR, the enhanced recombination process and counter-selection occur within a single bacterial cell (Figure 1E). Table 1 provides an overview of the existing literature on phage genome editing using various CRISPR-Cas systems. Among the 14 studies listed in the table, the majority (10 studies) employed the DPMSR strategy, which utilizes the CRISPR system to enhance the recombination process and achieve higher editing efficiency. Notably, the VI-A (Cas13a) CRISPR system, which targets RNA (Gootenberg et al., 2017), was utilized in two related studies employing the SPMAR strategy for counter-selection purposes (Adler et al., 2022; Guan et al., 2022). However, no significant differences in editing efficiency have been reported when using these two different strategies for DNA-targeting CRISPR systems. Zhang et al. successfully used both strategies to delete a 292 bp gene fragment in the bacteriophage genome using an exogenous CRISPR-Cas9 system in *V. natriegens* TT4. The editing efficiency obtained with both strategies was high and similar (Zhang X. et al., 2022).

**Table 1:** Previous studies of phage genome using the CRSIPR-Cas system.

CRISPR type	Host bacteria and bacteriophages	Mutations introduced	Editing efficiency	Genes substituted or inserted	Genome editing strategy	References
II-A^a^	*Streptococcus thermophilu*, 2,972	Point mutations Gene deletions Gene substitution	100% (10/10) 100% (10/10) NM^f^	The LlaDHCIA methyltransferase gene^c^	DPMSR^i^	Martel and Moineau (2014)
II-A^b^	*Escherichia coli*, T4	Point mutations Gene deletions	100% (20/20) 100% (5/5)	NM	DPMSR	Tao et al. (2017)
	*Lactococcus lactis*, P2	Gene deletion Point mutations Gene substitution	33% (1/3) NM NM	The polyhistidine-tag at the N-terminus of Orf47^c^	DPMSR	Lemay et al. (2017)
	*Bacillus subtilis*, Goe1	Gene deletions Gene substitution	41.6% (15/36) 5% (2/40)	The bgaB gene, coding for a thermostable beta-galactosidase from *Bacillus* stearothermophilus^d^	DPMSR	Schilling et al. (2018)
	*Listeria monocytogenes*, A511	Point mutations Gene substitution	NM NM	The lysostaphin gene (lysostaphin-his6)^d^	SPMAR^j^	Hupfeld et al. (2018)
	*Klebsiella pneumoniae*, phiKpS2	Point mutations Gene deletions Gene insertion	100% (30 bp, 40 bp)^g^ 100% (40 bp, 50 bp)^g^ 87.5% (60 bp)^g^	The gene for red fluorescent protein (rfp)^c^	DPMSR	Shen et al. (2018)
	*Vibrio natriegens* TT4, TT4P2	Gene deletions Gene insertion	97% (34/35, DPMSR) 100% (19/19,SPMAR) 75% (15/20, SPMAR)	The lysozyme e gene of phage EJ^d^	DPMSR or SPMAR	Zhang J. et al. (2022)
	*Escherichia coli*，T3, T5, and T7	Gene deletion Gene insertion Gene substitution	100% (20/20), (T3)^h^ 100% (7/7), (T7)^h^ 92% (11/12), (T5)^h^ 50–89% (T7)^h^	The EYFP tag in the T7 genome^c^	DPMSR	Isaev et al. (2022)
I-E^b^	*Escherichia coli*, T7	Gene deletions	38.6% (17/44) 41.6% (15/36)	NM	SPMAR	Kiro et al. (2014)
	*V. cholerae* El Tor，ICP1	Gene deletions Gene insertion	100% (8/8) 58% (7/12) 50% (4/8)	The gene for green fluorescent protein (gfp)^c^	DPMSR	Box et al. (2016)
III-A^a^	*Staphylococcus epidermidis* LAM104, Andhra and ISP	Gene substitution	100% (20/20)	The protospacer sequencing with several silent mutations^e^	DPMSR	Bari et al. (2017)
V^b^	*Escherichia coli*, T4	Gene deletions Gene insertion	100% (13/13) 100% (10/10) 100% (5/5)	The gene for green fluorescent protein (gfp)^c^	DPMSR	Dong et al. (2021)
VI-A^b^	*Escherichia coli*, T4	Gene substitution	100% (36/36)	The non-essential soc. gene or essential dnap using silent multiple continuous mutations^e^	SPMAR	Adler et al. (2022)
VI-A^a^	*Pseudomonas aeruginosa*, ФKZ, OMKO1, and PaMx41	Gene deletion Gene insertion	11.8–52.9% (ФKZ)^h^ 100% (PaMx41)^h^ 7–25% (ФKZ)^h^ 50–70.8% (OMKO1)^h^	The gene for mNeonGreen or mCherry^c^	SPMAR	Guan et al. (2022)

^a^Endogenous CRSIPR-Cas system. ^b^Heterologous CRSIPR-Cas system. ^c^Marker gene. ^d^Functional gene. ^e^Silent mutations. ^f^NM, not mentioned. ^g^The data in brackets represents the length of the homologous arm in the editing template. ^h^Names of genetically modified bacteriophages are in brackets. ^i^DPMSR, dual-plasmid-mediated synchronous recombination. ^j^SPMAR, single-plasmid-mediated asynchronous recombination.

Five types of CRISPR systems, namely II-A (CRISPR-Cas9), I-E (CRISPR-Cas3), III-A (CRISPR-Cas10), V (CRISPR-Cas12a), and VI-A (CRISPR-Cas13a), have been utilized for phage genome modifications (Table 1). The CRISPR systems II-A (CRISPR-Cas9) and I-E (CRISPR-Cas3) represent early-stage CRISPR systems that have undergone extensive research and development. They were among the first CRISPR systems employed for gene editing in phages. The CRISPR-Cas3 system involves multiple genes and proteins that interact with each other (Yosef et al., 2015), and it is characterized by low efficiency and operational challenges. Consequently, research on its application for phage gene editing remains limited. Kiro et al. introduced the Type I-E CRISPR-Cas System into *Escherichia coli* for engineering bacteriophages using the counter-selection method (SPMAR), resulting in a relatively low editing efficiency of 17 out of 44 plaques exhibiting the desired gene deletion (Kiro et al., 2014). In contrast, Box et al. introduced a combination of the Type I-E CRISPR-Cas System and a homologous recombination template on a single plasmid into *V. cholerae*. This approach led to successful gene deletion (Editing efficiency: 7/12) and gene replacement (Editing efficiency: 4/8 relevant genes in the ICP1 bacteriophage genome) (Box et al., 2016). The utilization of the Type I-E CRISPR-Cas system in these two cases did not yield high editing efficiency, possibly due to the more complex composition of the CRISPR-Cas system. In comparison, Cas9 demonstrates superior gene-targeting efficiency, cost-effectiveness, and ease of use compared to CRISPR-Cas3 (Lu et al., 2022). As a result, Cas9 is the most commonly employed CRISPR system for editing bacteriophage genomes. Martel and Moineau (2014) first demonstrated that the endogenous CRISPR-Cas II-A system in the thermophilic bacterium *Bacillus subtilis* could selectively pressure and modify specific point mutations, large deletions, and gene exchange in the genome of virulent bacteriophage 2,972, thereby enhancing recombination efficiency. Following this, researchers introduced heterologous CRISPR-Cas type II-A systems into various host bacteria, including *Escherichia coli* (Tao et al., 2017), *Klebsiella pneumoniae* (Shen et al., 2018), *Bacillus subtilis* (Schilling et al., 2018), *Vibrio natriegens* (Zhang J. et al., 2022), and *Listeria monocytogenes* (Hupfeld et al., 2018), to enable specific phage fragment shearing. In summary, the CRISPR-Cas9 system has exhibited broad applicability in bacteriophage gene editing and has consistently demonstrated high editing efficiency across the majority of the cases listed in Table 1.

The primary challenge in editing bacteriophage genes using the commonly used CRISPR-Cas9 system lies in the development of bacteriophage resistance. Bacteriophages can acquire resistance to the CRISPR-Cas9 system through various mechanisms, including the emergence of escape mutants and hindering attachment (van Houte et al., 2016). To overcome this challenge, it is important to explore alternative CRISPR systems that are better suited to tackle the resistance mechanisms exhibited by bacteriophages. The immune response generated by the CRISPR-Cas9 system in bacteria against phages often leads to the generation of escape mutants, which is not desirable in phage genome editing. However, the III-A type system offers a potential solution as it does not require a PAM (Protospacer Adjacent Motif) or a seed sequence, thereby preventing wild-type phages from evading detection through single nucleotide replacements. This feature makes the III-A type system a promising approach to counter the emergence of escape mutants and enhance the effectiveness of phage genome editing. Bari et al. utilized the endogenous CRISPR-Cas10 system of *Streptococcus pneumoniae* to introduce targeted fragments and editing templates into bacterial cells, conducting bacteriophage gene engineering during the infection of the transformed bacteria. This approach confirmed a relatively high editing efficiency of 10/10 (Bari et al., 2017). The impeded attachment of phage nucleic acid protects it from being targeted by the CRISPR system. Choosing a suitable CRISPR system can counteract the protective effect of impeding attachment in some cases. Recently, Dong et al. introduced exogenous Type V CRISPR and Type II CRISPR-Cas9 into *Escherichia coli* separately to modify T4 phage. Their findings indicate that the T4 bacteriophage genome is modified by ghmc, conferring resistance to Type II CRISPR-Cas9 systems. The efficiency of the Cas12a editing system depends largely on crRNA, and the Cas12a system can effectively cleave the ghmc-modified genome to generate double-stranded DNA breaks (DSBs), facilitating efficient recombination between the phage DNA and the donor plasmid. The gene editing efficiency achieved in the experiment was 100% with the participation of Cas12a (Dong et al., 2021).

Recently, Type VI (CRISPR-Cas13) system has been employed for genome editing of bacteriophages (Adler et al., 2022; Guan et al., 2022). Type VI system can be utilized for RNA-targeted modification and regulation. During bacteriophage infection, the Type VI CRISPR system can remarkably induce host dormancy, effectively inhibiting the emergence of escape mutants (Meeske et al., 2019). Therefore, the Type VI system represents a highly advantageous tool for counter-selection when employing the SPMAR strategy. In support of this, Adler et al. conducted a study where they introduced Cas13a into *E. coli* to facilitate the counter-selection of modified bacteriophages in the progeny. Remarkably, all the phages selected through this screening process were subsequently confirmed to be modified as intended. The targeting of Cas13a also lacked the PAM requirement, indicating that virtually any position within or near the phage transcript can be edited and selected by Cas 13a counter-selection (Adler et al., 2022). Therefore, employing Type VI systems for counter-selection of modified bacteriophages is a precise and effective approach.

Among the different systems, CRISPR-Cas9 is the most commonly used due to its high efficiency and versatility. However, in cases where the CRISPR-Cas9 system is ineffective against certain phages, alternative CRISPR systems, such as Cas10 (Bari et al., 2017), Cas12 (Dong et al., 2021) have been explored. Additionally, Type VI system is a favorable tool for counter-selection using the SPMAR strategy. The use of CRISPR-Cas in phage genome editing relies on the innate or exogenous defense system of bacteria to target the wild-type gene fragment. However, bacteriophages possess the remarkable ability to develop resistance to the immune response induced by specific CRISPR systems. This resistance can arise through mechanisms, such as escape mutations or impeding attachment, leading to a decrease in the efficiency of gene editing. Therefore, it is crucial to understand the different characteristics of CRISPR systems to select the appropriate one based on the specific situation to achieve the desired editing effect.

### 2.3. The study of retron system and its application in phage gene editing

Retrons are a type of bacterial retroelements that generate single-stranded reverse transcribed DNA (RT-DNA) and are composed of three essential components: the reverse transcriptase RT, a non-coding RNA (ncRNA), and one or more accessory proteins (Millman et al., 2020; Bobonis et al., 2022). Despite being discovered more than three decades ago, the function of retrons has remained elusive (Lampson et al., 1989). However, recent studies have shed light on some of the mechanisms and potential applications of retrons, which is an exciting development in this field.

Retrons are often found in the defense islands of bacteria (Hampton et al., 2020) and have been identified as important players in bacterial defense systems (Millman et al., 2020). The auxiliary protein RecB is a critical component of this defense system. The RT-ncRNA complex binds to RecB and acts as a “guard” to monitor its activity (Millman et al., 2020). When a phage protein invades and directly targets RecB, the RT-ncRNA complex detects this change, signaling the onset of a phage infection (Millman et al., 2020). This triggers the activation of the abortive infection mechanism. Some retrons are not found in bacterial defense islands or alongside known defense systems, and they appear to be adapted to perform functions other than phage defense, similar to the toxin-antitoxin system. Some retrons are involved in anti-phage defense, while others play a role in the bacterial stress response (Harms et al., 2018). Recent studies have reported the discovery of tripartite toxin-antitoxin systems composed of retron elements containing RcaT (Bobonis et al., 2022). RcaT-containing retrons are the most prevalent retron family in bacteria and have a high prevalence in proteobacteria (Mestre et al., 2020). RcaT encodes an auxiliary toxin protein that can form tripartite toxin-antitoxin systems (TAs) with the RT-msDNA antitoxin complex (Bobonis et al., 2022). Unlike the retrons systems containing RecB, phage-introduced trigger factors directly modify the msDNA, disrupting the stability of the TAs system and activating the RcaT toxin protein, leading to abortive infection (Millman et al., 2020; Bobonis et al., 2022). Based on these latest findings, it can be deduced that during the retron system’s defense against bacteriophage invasion, certain bacteriophage proteins can interact directly with auxiliary proteins or msDNA in the retron components, resulting in abortive infection and preventing bacteriophage replication and spread. To understand which phage proteins are involved in the defense system of retron, as these proteins impede the production and amplification of engineered phages, high-throughput methods can be utilized to identify these phage proteins.

In the absence of a natural manipulator, the expression of ncRNA and RT from plasmids lacking accessory proteins constitutes the minimal system for RT-DNA production. This system can be utilized for in-cell template gene amplification, enhancing the efficiency of gene editing (Sharon et al., 2018; Simon et al., 2019; Lopez et al., 2022). Furthermore, the RT-ncRNA system can be integrated with CRISPR-Cas for molecular tagging. In this process, the mRNA transcribed from the gene with the tagged promoter is reverse-transcribed into RT-DNA, which is then integrated into the host cell’s CRISPR array in sequence under the action of the CRISPR-Cas integrase, creating a permanent transcription record (Bhattarai-Kline et al., 2022).

Retrons have shown promise as a tool for gene editing in various studies, but the development of scalable retron-based gene editing technologies remains a challenge due to a limited understanding of retrons, particularly their retrotransposition mechanisms. The most commonly used retron in these studies is the engineered Ec86, also known as Eco1, which was the first retron discovered in *E. coli* (Wang et al., 2022). Retron engineering typically involves modifying non-coding RNAs (ncRNAs). For example, the msd sequence in Ec86’s ncRNA, which is reverse transcribed, can be replaced and used as a template for homology-directed repair (HDR) (Sharon et al., 2018). Additionally, extending the length of the a1/a2 complementary region in the msr sequence, which remains RNA in the final molecule and partially overlaps, can increase reverse transcriptase-mediated DNA production (Lopez et al., 2022). Cryo-electron microscopy (cryo-EM) analysis of Ec86 retron complexes in *Escherichia coli* has revealed that reverse transcriptase serves as an anchor point for auxin, facilitating direct interaction between msDNA and auxin (Wang et al., 2022). It may be a development trend to optimize the genome editing system based on transcriptase and msDNA structure for Retron-based gene editing.

Retron-based techniques for editing phage genes require careful consideration of various factors, such as the design of the template DNA in the msd sequence and the ability of modified transcripts to still mediate phage defense. To address these issues, Rossier et al. introduced an engineered Ec86 retron carrying a DNA template fragment into wild-type *Escherichia coli*, taking advantage of the bacterium’s reverse transcription capabilities to attain high concentrations of the editing template and facilitate homologous recombination (Figure 1D), resulting in the generation of T5 mutant phages. However, the editing efficiency of the phage genome editing using retron was suboptimal at 2% (2/100) (Ramirez-Chamorro et al., 2021), indicating a need for further optimization to improve the technique. Despite this, the editing template provided by the retron element offers several advantages over other methods of homologous editing templates. First, there is no need for targeted mutation of the cloned fragment, as the small size of the retron enables the cloning of annealed oligonucleotides carrying the desired mutation. Second, unlike classical HR templates, the retron-based approach does not require long sequences on either side of the mutation, which may encode toxic products for the host cell (Ramirez-Chamorro et al., 2021).

## 3. Phage synthesis outside the host bacterium

**Figure 2:**
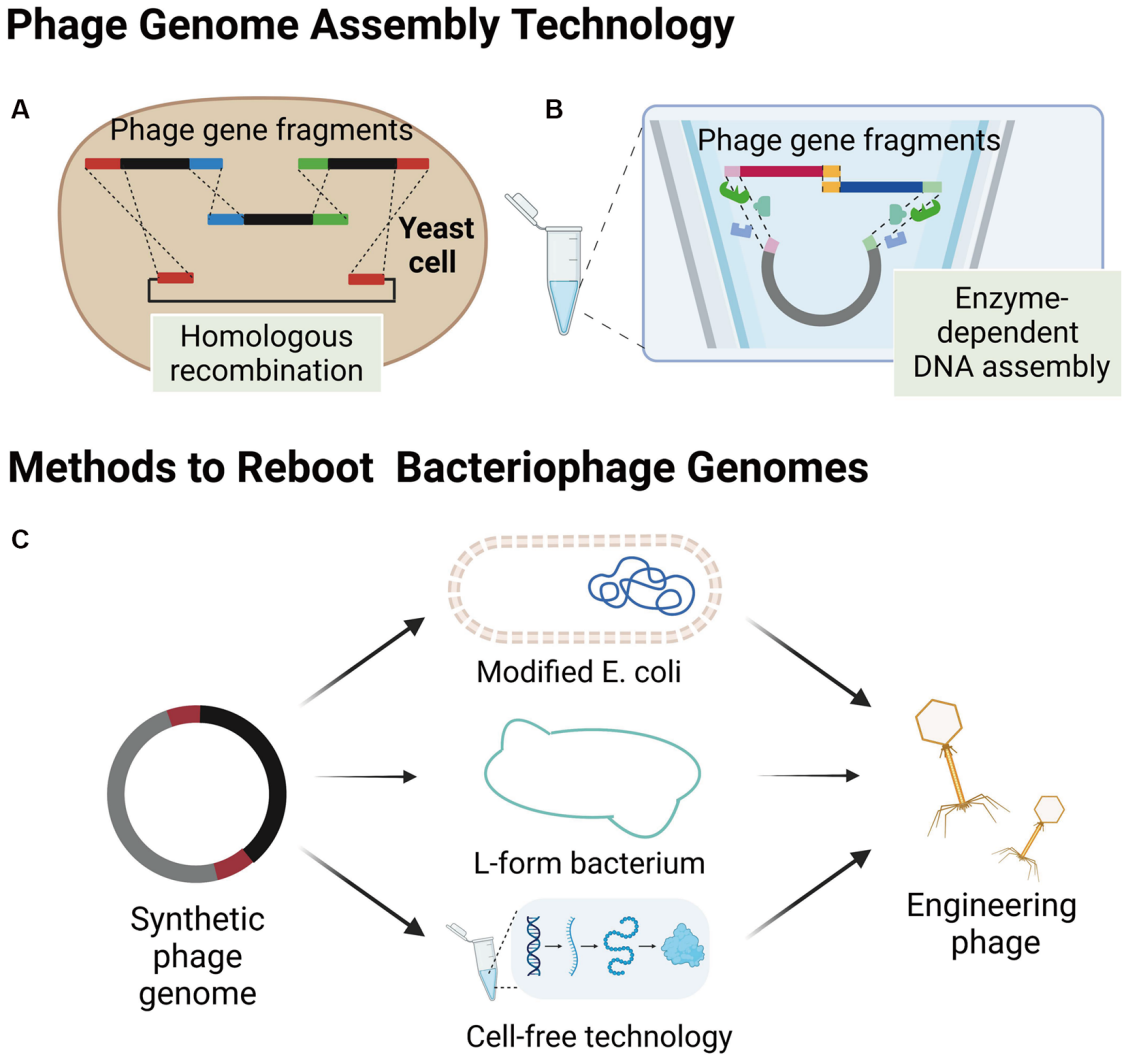
Assembly of bacteriophages outside host bacteria. **(A)** Smaller phage gene fragments are assembled in yeast cells by homologous recombination. **(B)** Enzyme-dependent DNA assembly, a cell-free genome assembly method for bacteriophage that utilizes exonucleases, polymerases, and ligases. **(C)** Several methods of engineering bacteriophage reboot (images are created with Biorender.com).

The process of synthesizing bacteriophages outside of their host bacteria involves the synthesis of the bacteriophage genome in alternative environments, including yeast cells or *in vitro*. Subsequently, the synthesized genome is transferred into an appropriate environment, such as a modified *E. coli*, L-form bacterium, or a cell-free TXTL platform, to initiate the engineered bacteriophage production (Figure 2). This approach offers several advantages for genetic modifications as the process occurs independently of the host bacteria. Consequently, it allows for a high degree of customization and facilitates more efficient genetic modifications without the need for extensive screening of engineered bacteriophages. Additionally, synthesizing bacteriophages outside of their host bacteria overcomes the challenge posed by the poor transformation capabilities of the host bacteria, which often hampers the editing process within the host bacteria. By utilizing alternative environments, scientists can optimize the genetic modification process, thereby opening up new possibilities for enhancing the capabilities and applications of bacteriophage engineering.

### 3.1. Gene assembly *in vitro*

The yeast artificial chromosome (YAC) is a versatile tool for manipulating the design and construction of targeting vectors (Murray, 2006), as well as for assembling phage genomes. Small phage gene fragments can be assembled in yeast cells using transformation-associated recombination (TAR cloning) of complete DNA molecules (Figure 2A; Noskov et al., 2001; Gibson et al., 2008). Additionally, Enzyme-dependent DNA assemblies, such as the Gibson assembly and Golden gate assembly have been extensively used for synthesizing phage genomes *in vitro* (Figure 2B; Gibson et al., 2008, 2009; Kilcher et al., 2018). The *in vitro* synthesis approach provides a highly efficient and precise method for modifying specific gene sequences within bacteriophage genomes. Notable examples of this approach include the modular assembly and replacement of RBPs in bacteriophage genomes using yeast cell systems (Ando et al., 2015), as well as the construction of mutated bacteriophage gene libraries through golden gate assembly (Liang et al., 2022). One of the major advantages of *in vitro* synthesis is its superior efficiency compared to intracellular recombination within host cells. Furthermore, the rebooting of bacteriophages using synthesized genomes eliminates the need for phage screening and subsequent cloning, thereby significantly increasing the overall efficiency of engineering bacteriophages (Ando et al., 2015; Liang et al., 2022). By harnessing the power of *in vitro* synthesis, researchers can achieve precise and targeted modifications in bacteriophage genomes, paving the way for advancements in the field of bacteriophage engineering.

### 3.2. Reboot bacteriophages in non-host bacteria

Electroporation has demonstrated a high transformation efficiency of *E. coli* (Marinelli et al., 2008, 2012). Thus, using electroporation for the transformation of synthetic genes of phages hosted by *E. coli* and other bacteria into *E. coli* for rebooting is a feasible approach. The success of this approach relies on whether the transcription and translation systems in *E. coli* meet the requirements for phage gene rebooting. To date, this method has only been reported to work with gram-negative phages, including *E. coli* phage phiX174 (Jaschke et al., 2012), T7-like (Ando et al., 2015), S13-like (Liu et al., 2012), and the large *Salmonella* Myovirus FelixO1 (Kilcher and Loessner, 2019). Recently, Cheng et al. developed the *E. coli* strain DH10B as a “stepping stone host” for phage rebooting and demonstrated its capability of reactivating 126 phages from T7 and non-T7 families that infected clinical multi-drug resistant *Klebsiella pneumoniae*, *Streptococcus enterica*, *Pseudomonas aeruginosa*, and *Pseudomonas baumannii*, indicating its potential application for the production of phages targeting drug-resistant bacteria (Cheng et al., 2022). Additionally, DH10B strains can uptake large amounts of DNA and protect exogenous DNA from restriction systems (Durfee et al., 2008), which enables Cheng et al. to transform bacteriophage DNA fragments into DH10B for bacteriophage assembly and reboot.

Gram-positive bacteria are challenging to transform due to their thick peptidoglycan layer, making it difficult to transform the phage genome. However, researchers have overcome this issue by generating L-form bacteria through long-term cultivation of gram-positive bacteria in the presence of cell wall-activating compounds, such as penicillin, which destroys the cell wall (White et al., 1981). This type of cell wall-deficient bacteria is more conducive to the transformation of large DNA molecules and is suitable for rebooting the corresponding bacteriophages. Furthermore, L-form bacteria with cell wall defects are susceptible to being permeabilized, releasing the engineered bacteriophages when subjected to dilution in an unstabilized medium (Kilcher et al., 2018).

L-form bacteria, which are characterized by their lack of a cell wall, present a unique advantage in the field of phage genome engineering. Their cell wall-deficient nature renders them highly susceptible to the uptake of foreign DNA and phage genomes, facilitating the genetic manipulation of these bacteria (Kilcher et al., 2018). Furthermore, the ability to cultivate L-form bacteria in liquid cultures offers a significant improvement in efficiency and speed compared to the conventional method of growth on solid media. This feature enhances the feasibility and effectiveness of rebooting phage genomes, making L-form bacteria a powerful tool in phage engineering research. Kilcher et al. (2018) also developed an L-type *Listeria* bacterium called Rev2L, which has been shown to reactivate eight *Listeria monocytogenes* phages, two *Bacillus* phages, and two *Staphylococcus aureus* phages, including mild (B025, B035, B056, PSA) and virulent phages (P35, P70, P100, A511), some of which have large genomes (>130 kb; P100, A511). These results demonstrate the versatility of the L-type bacterium as a reboot host.

### 3.3. Reboot of phage in a cell-free environment

To successfully reboot bacteriophages in a cell-free environment, it is essential to understand the mechanism of bacteriophage DNA expression within bacterial cells. When phage DNA enters the host cell, it hijacks the bacterium’s transcriptional system to express the associated enzymes in the phage gene, resulting in the generation of progeny phages and lysis of the host bacterium (Yang et al., 2014; Gogarten, 2022; Liao et al., 2022). The transcription of phage DNA in the host cell is divided into three stages: early, middle, and late (Melo et al., 2022). In the early stage, phage DNA expresses a set of genes responsible for hijacking the host transcription machinery, including the RNA polymerase, which is used by the original RNA polymerases in the host cell (Yang et al., 2014). After gaining control over the host, phage DNA expresses DNA enzymes that break down host cell DNA, DNA polymerase for replicating viral DNA, HMC synthetase, and mRNA polymerase for subsequent steps. During the final stage, a significant number of “components” are generated through mRNA translation, including head protein, tail protein, various assembly proteins, and lysozyme, which facilitate the assembly of progeny viruses (Roucourt and Lavigne, 2009).

The transcription mechanism of bacteriophages varies depending on the presence or absence of endogenous RNAP. For bacteriophages that lack endogenous RNAP, such as bacteriophage T4 and λ, gene expression is regulated through the encoding of specific protein factors, such as bacteriophage P23–45 gp39 protein (Berdygulova et al., 2011) and bacteriophage λ N protein (Archer et al., 2014), that modulate host RNAP specificity. In contrast, bacteriophages with endogenous RNAP, such as T7 and Xp10, utilize the host *E. coli* RNAP to transcribe early genes, but then switch to their own RNAP to activate middle and late promoters. Additionally, some small proteins of the bacteriophage inhibit the function of the host RNAP, such as T7 gp2 and Xp10 P7. Thus, protein factors encoded by bacteriophages play a critical role in bacteriophage expression. They alter RNAP specificity or function by interacting with RNAP, thereby transferring cellular resources to bacteriophage gene expression.

Our investigation focuses on the gene expression process of bacteriophages within bacteria and the involvement of host proteins, which are fundamental to our approach in restarting bacteriophages *ex vivo*. To achieve this, we rely on the cell-free gene expression (CFE) system, an essential tool for investigating these matters. The CFE system utilizes cell extracts to activate transcription and translation, preserving the native transcription and translation machinery of cells (Silverman et al., 2020). We can initiate protein synthesis *in vitro* using the CFE system by supplementing the extracts with exogenous resources, such as amino acids, nucleotides, and secondary energy substrates (Silverman et al., 2020). With the combined use of CFE and proteomics mass spectrometry, we can investigate the contents of various bacteria, particularly the gene regulatory elements, such as promoters and transcription factors. The transcriptional mechanisms of several bacteria, including *Bacillus megaterium* (Moore et al., 2018), *Pseudomonas putida* (Wang et al., 2018), *Clostridium autoethanogenum* (Kanehisa et al., 2020), and *Vibrio natriegens* (Bjorling et al., 2018; Djokic et al., 2018; Shen et al., 2018), have been investigated using this approach, leading to the development of corresponding CFE platforms. Moreover, Yim et al. (2019) used the Cell-Free Transcription and Sequencing (DRAFTS) method to investigate gene transcription activity in the cell-free lysates of 10 different bacteria from three phyla (Proteobacteria, Firmicutes, and Actinobacteria). They found both commonalities and differences in the transcriptional mechanisms of different bacteria, prompting the use of non-host-derived CFE systems or mixed CFE systems from multiple host sources to produce corresponding bacteriophages.

CFE can be utilized to directly examine bacteriophage transcription and translation. Emslander et al. employed a combination of CFE technology, time-resolved mass spectrometry, and plaque assay with T7 bacteriophage as verification, resulting in consistent expression of early, middle, and late-stage genes as previously proposed (Emslander et al., 2022). This approach is valuable for investigating the process, which can help improve the production of CFE.

The utilization of CFE technology in bacteriophage genome editing offers several advantages. Firstly, it eliminates the necessity of screening for engineered bacteriophages and assessing transformation efficiency. Instead, the emphasis shifts toward bacteriophage reboot, streamlining the editing process. This has led to increased attention toward cell-free bacteriophage synthesis, especially with the rapid advancements in CFE technology. Simple protein-based viral structures, such as phages, have been employed to validate cell-free biomanufacturing through *in vitro* transcription and translation of the entire viral genome. These efforts resulted in self-replicating phages, including *E. coli* phage T4 (Rustad et al., 2018), T7 (Shin et al., 2012), ФX174 (Rustad et al., 2017), phiX174 (Garamella et al., 2016), MS2 (Rustad et al., 2017), and K1F (Liyanagedera et al., 2022). Emslander et al. (2022) extended cell-free production to phages targeting the Gram-positive bacterium *Bacillus subtilis* by co-expressing appropriate host factors. In this example, highly similar RNA polymerases were utilized in both *Escherichia coli* and *Bacillus subtilis*.

In summary, the successful synthesis of bacteriophages through cell-free methods hinges on the proper assembly and reboot of the specific bacteriophage being studied. This entails investigating the transcription and translation systems of the bacteriophage, as well as the influence of host factors on the reboot process. The use of cell-free extracts (CFE) offers a means to acquire this knowledge and enhance CFE production efficiency. The development of CFE technology has made significant strides, with standardized methods for preparing extracts capable of transcription and translation from various bacteria having been established (Silverman et al., 2020). Furthermore, alternative CFE systems sourced from organisms other than *Escherichia coli* have been developed, indicating the feasibility of using different platforms for bacteriophage production with non-model hosts (Bjorling et al., 2018; Moore et al., 2018; Shen et al., 2018; Wang et al., 2018; Kanehisa et al., 2020; Xu et al., 2020).

## 4. Modification of bacteriophage receptor binding proteins

The majority of phages that have been described so far have a tail morphology and contain a double-stranded DNA genome ranging from 15 to 500 Kbp, belonging to the order Caudovirales (Ackermann, 2007; Malik, 2019; Dion et al., 2020). These tail phages consist of a head that is made up of an icosahedral capsid and a tail that interacts with the surface receptors of host bacteria through surface receptor proteins (RBPs) (Malik, 2019). The initial step in bacteriophage infection of host bacteria is the binding of the RBPs to specific receptors present on the surface of bacteria. This interaction plays a vital role in guiding subsequent steps of the infection process and determining the range of host bacteria that the bacteriophage can infect (Chen et al., 2020; Wei et al., 2022).

### 4.1. Identifying receptor binding proteins and host receptors

Phage RBPs can be classified as tail fibers (TF) or tail spikes (TSP) based on their structural features. Morphologically, both TFs and TSPs consist of homotrimeric complexes, with TFs forming long, thin filaments that interact with protein and/or sugar receptors, whereas TSPs form more globular complexes (Klumpp et al., 2023). TFs are elongated fibrillar proteins lacking enzymatic activity and contain specific receptor binding sites at their distal ends that are involved in host binding. Besides, TSPs are shorter and more rigid with enzymatically active globular structures at their terminal ends, which are usually active against specific surface structures (such as the sugar fraction, phosphopeptides in Gram-positive hosts or lipopolysaccharides, and capsule polysaccharides in Gram-negative hosts) (Dunne et al., 2021; Klumpp et al., 2023). The presence of RBPs varies depending on the morphological characteristics of bacteriophages. For instance, Myoviridae phages (with icosahedral heads, retractable tails, and six short spines/long flagella that infect Gram-negative bacteria) and Siphoviruses (polyhedral heads with elongate, non-retractable tails, and short fibers, McCorquodale, 1999) typically have TFs as RBPs (Letarov and Kulikov, 2017). In contrast, TSPs are the more common RBPs in Podoviridae phages (with diverse polyhedra and short, non-contractile tails, Molineux, 1999; Letarov and Kulikov, 2017; Gonzalez and Scharf, 2021).

Phage receptor binding proteins (RBPs) play a critical role in initiating the infection process by binding to specific receptors on the surface of host bacteria. The receptors in Gram-negative bacteria are primarily lipopolysaccharides (LPS), outer membrane proteins (OMPs), and fimbria or capsule components, whereas Gram-positive bacteria have cell wall teichoic acid (WTA), lipoteichoic acid (LTA), and cell wall-associated polysaccharides as their primary receptors for phage RBPs (Ha et al., 2019). Recent studies on the receptors of bacteriophages for *Escherichia coli* or related gut bacteria have shown that Siphoviruses target porin proteins on the cell surface, Podoviridae phages target polysaccharides, and Myoviridae phages can target either of these two (Letarov and Kulikov, 2017). These differences in cell wall composition, thickness, lipid and lipoprotein content, and receptor specificity suggest that phages have evolved to infect specific bacterial hosts, highlighting the importance of understanding the molecular mechanisms underlying phage-host interactions.

The introductory information provides a general understanding of the RBPs in phages and bacterial receptors, which aids in the screening of phage RBPs for subsequent research. However, for unknown novel phages, bioinformatics methods are required to explore the RBPs of phages. The continuous mutation of phage RBPs genes resulting from the arms race between phage tails and host surface receptors has been extensively researched (Lee et al., 2016; Yuan et al., 2019). Moreover, in certain natural environments, the exchange of RBPs genes between different types of phages occurs, causing changes in host ranges to adapt to changing environments (Xiao et al., 2018; Górniak et al., 2022). These two naturally occurring processes result in some degree of homology between the RBPs of phages corresponding to the same or different host bacteria. Bioinformatics methods use this homology to help predict the RBPs of phages. In our study of the phage genome, we can select genomic annotation of TFs, TSPs or related genes that are vaguely annotated as “tail proteins” or “substrate proteins” for subsequent research (Boeckaerts et al., 2021; Phetruen et al., 2022). Furthermore, homology modeling of genes with high homology can be performed, selecting models with high coverage and confidence in the target sequence to analyze possible docking structural domains and associated sites (Yehl et al., 2019). The RBPs predicted through bioinformatics analysis can then be validated using various *in vivo* and *ex vivo* methods, such as affinity chromatography, knockout mutagenesis, phage plaque assays, and phage adsorption kinetic analysis (Chen et al., 2020).

Once bacteriophage RBPs are identified, we can engineer modifications to meet host range requirements, which mimic interactions between bacteriophages and bacteria in nature. Table 2 presents a synopsis of cases over the past 8 years in which bacteriophages were modified using evolutionary methods, phage chimeric methods, and RBPs gene swapping. The methods and outcomes of each study are briefly outlined in Table 2, and the following sections will elaborate on the significance of each approach.

**Table 2:** Engineered modification of bacteriophage RBPs.

Methods	Phage and its modification site (source of replacement/inserted gene)	Results	Host receptor (host bacteria name)	References
Evolution	*E. coli* bacteriophage T3; gp17	Mutating HRDRs yields phagebodies with altered host-ranges, and select phagebodies enable long-term suppression of bacterial growth *in vitro*	LPS (*E. coli*)	Yehl et al. (2019)
	*E. coli* bacteriophage T4; gp37	Phage mutants can adsorb lipopolysaccharide of OmpC or K-12 strain of O157 strain	OmpC (*E. coli*)	Suga et al. (2021)
	*V. cholerae* bacteriophage ICP2; gp25	Single or double mutations in gp25 are sufficient to generate the host range mutant phenotype, but to stably infect specific OmpU mutant strains, an additional mutation must be introduced in gp23	OmpU (*V. cholerae*)	Lim et al. (2021)
	*E. coli* bacteriophage T7; gp17	Phage mutants can infect *Yersinia enterocolitica*, a wild-type T7 phage that cannot be lysed	NM^a^	Liang et al. (2022)
	*Listeria* bacteriophage PSA; gp15	Multivalent phages with two RBPs generated true heterocapsids with similar efficiency in infecting the *SV 4b* strain WSLC1042 as the wild-type phage and showed loose specificity toward the GlcNAc sugar-deficient isogenic derivative of WSLC1042	Galactosylated wall teichoic acid (*Listeria*)	Dunne et al. (2019)
Chimeric phage tail fibers	Bacteriophage T2; DenA, α-gt, gp12 and gp37–38(those genes in bacteriophage PP01 that can infect *E. coli* O157: H7)	The modified T2 phage has improved the adsorption capacity of *E. coli* O157: H7, but they show difficulties in infecting bacteria	NM	Hoshiga et al. (2019)
	*E. coli K12* bacteriophage T7; gp17 (gp17 in bacteriophage K1F that can infect *E. coli* K1)	The reduced repeatability of phage replication in the new host (*E. coli* K1), unable to promote sustained autonomous replication	NM	Avramucz et al. (2021)
	*E. coli* bacteriophage P2; *gph* (gp37 of *Salmonella* bacteriophages S16)	Pseudotyped P2 can be significantly retargeted to *Salmonella* via OmpC	P2b-LPS (*E. coli*) S16-OmpC (*Salmonella*)	Cunliffe et al., 2022
	Bacteriophages STyj5-1; PB1 (PB1 of wide-host range bacteriophage BD13)	In comparison to the wild type, the engineered phage displays a broader host range and a higher rate of absorption	NM	Zhang J. et al. (2022)
	*S. flexneri 2,457 T* bacteriophage P2; tail proteins gene H, gene G (the tail proteins of *S. flexneri* bacteriophages P1(S′) that can infect *S. flexneri M90T*)	Chimeric tail P2-P1(S′) improved the bacteriophage sensitivity to transduction efficiency in *S. flexneri* M90T	P2-OmpC (*S. flexneri* 2,457 T) P1(S′)-O-antigen and core LPS (*S. flexneri* M90T)	Fa-Arun et al. (2023)
Swapping receptor binding genes	*E. coli* bacteriophage T3; gp17 (*Yersinia pseudotuberculosis* bacteriophage phiA1122 TF gene); *E. coli* bacteriophage T7; gp11, gp12, gp17 (gp11, gp12, and gp17 of *Klebsiella K11* bacteriophage)	The modified T3 phage can infect *Yersinia pseudotuberculosis*; The modified T7 phage can infect the synthetic phage of *Klebsiella* K11	T3-the penultimate glucose residue of the LPS (*E. coli* B strains), phiA1122-the core LPS (*Yersinia*) T7-an undetermined site further inward in the LPS (*E. coli B strains*) K11 phage-the capsule (*Klebsiella* K11)	Ando et al. (2015)
	*E. coli* bacteriophage T7; gp11, gp12, gp17 (tails gene from various sources)	This study revealed the connection between DNA and mutations in the required tail-coding genes, and identified phages with higher transduction efficiency compared to the wild type.	NM	Yosef et al. (2017)

^a^NM, Not mentioned. ^b^Name of phage corresponding to the host receptor.

### 4.2. Evolutionary methods for modifying receptor recognition genes

By utilizing evolutionary methods to modify bacteriophage RBPs, particularly the host range determining regions (HRDRs), it is possible to alter the host range and infectivity of bacteriophages. The method of evolution involves naturally evolved methods and artificially introducing mutations in the RBPs region of bacteriophages (Yehl et al., 2019; Lim et al., 2021). Naturally evolved methods refer to the repeated cultivation of wild-type phages with mutant strains, which promotes phage evolution. Artificially induced mutations are generated *in vitro* through the use of degenerate or random primers (Yehl et al., 2019), error-prone PCR (Dunne et al., 2019; Suga et al., 2021), and golden gate assembly (Liang et al., 2022) to create a random mutation library. The formation of mutant bacteriophages in RBPs is achieved through homologous recombination (Yehl et al., 2019; Lim et al., 2021; Suga et al., 2021), which introduces genes from the mutant library into RBPs, or by rebooting the synthesis of mutant bacteriophages outside the host, e.g., rebooting in L-form bacteria (Dunne et al., 2019) or cell-free platforms (Liang et al., 2022). The mutated bacteriophage is then screened using different host libraries to obtain the engineering bacteriophage. This method has been used to modify the host range of phages. For example, the T4 bacteriophage with gp37 mutation can attach to hosts, such as the K-12 strain of *E. coli* O157 strain, which has OmpC on its surface (Suga et al., 2021), and the *Listeria* phage PSA with gp15 mutation can infect SV 4d strain *WSLC1033*, which lacks WTA galactosidase function (Dunne et al., 2019). These are characteristics that are lacking in wild-type phages but can be acquired through evolutionary means. The research by Yehl et al. (2019) is significant because they investigate the phage-host attachment process from a more nuanced perspective. They generated substantial diversity through the substitution of every codon in the target RBPs’ ring with a random codon, resulting in the completely randomized DNA-level sequence of the RBPs gene. This was then screened through corresponding bacterial libraries to obtain phages with desired host ranges. This approach allows for the exploration of the relationship between the physical and chemical properties of each loop region in the receptor recognition area and the host range by using host libraries composed of different phenotypic bacteria to identify the infectivity of mutated phages. Yehl’s method is suitable for engineering modification of minor host range changes, especially in cases where wild-type phages have been rendered resistant by the bacterium, as it can quickly produce phages that are resistant to host bacterium resistance (Yehl et al., 2019).

Through multiple-site mutations and proper experimental design, evolutionary methods can also help us investigate the molecular mechanism by which phage RBPs determine host range. Lim et al. (2021) introduced mutations in gp23 and gp25 in the *V. cholerae* bacteriophage ICP2, either separately or simultaneously. They found that single or double mutations in gp25 were sufficient to produce host-range mutant phenotypes, but to stably infect specific OmpU mutant strains, an additional mutation must be introduced in gp23. Mutation in gp23 alone was insufficient to produce host-range mutation phenotypes.

### 4.3. Chimeric methods in bacteriophage (RBPs) engineering

Chimeric methods in bacteriophage (BRP) engineering can be utilized to enhance the host range of modified bacteriophages. This involves combining the tail proteins of different types of bacteriophages that share high homology. This approach can lead to a more significant expansion of the host range than evolutionary methods alone. For instance, one study integrated the *E. coli* bacteriophage ϕV10 into the tail of the *S. flexneri* bacteriophage T2, allowing T2 to transduce the non-host bacterium *E. coli* O157:H7 (Hoshiga et al., 2019). Another study indicated that the insertion of *Salmonella* bacteriophage S16 gene gp37 into the TF gene (gph) of *E. coli* bacteriophage P2 resulted in the redirection of host bacteria from *E. coli* to *Salmonella* (Cunliffe et al., 2022). However, chimeric receptor binding proteins (RBPs) may not guarantee stable infection of target bacteria. In a study conducted by Avramucz et al., the gp17 gene (TF gene) from bacteriophage K1F, which targets *E. coli* K1, was inserted into the gp17 gene of bacteriophage T7, which targets *E. coli* K12. The resulting engineered phage exhibited unstable infectivity toward *E. coli* K1, leading to insufficient replication capacity (Avramucz et al., 2021). Similarly, Hoshiga et al. (2019) integrated several RBPs genes from *E. coli* bacteriophage PP01 into the corresponding genes of bacteriophage T2 of *E. coli*. Although the engineered phage T2 demonstrated effective adsorption to *E. coli*, successful infection proved challenging (Hoshiga et al., 2019).

### 4.4. Swapping receptor binding genes

The host range of phages is directly influenced by the receptor recognition genes, making it possible to alter the host range by swapping the entire receptor gene of a phage. *In vitro* synthesis of phage genomes offers a practical approach for such gene swapping. Ando et al. (2015) developed a yeast-based platform for the production of synthetic phage genomes, assembled from fragments (Ando et al., 2015). This innovative approach enables the exchange of receptor-binding proteins (RBPs) and tail components among different phage scaffolds. To test the modification capabilities of the platform, Ando et al. (2015) utilized two *E. coli* phages, T3 and T7. By replacing the receptor recognition genes (gp17) of T3 bacteriophage with those from Yersinia phage TFs, they successfully engineered a T3 bacteriophage capable of infecting *Yersinia pseudotuberculosis*. Furthermore, they replaced the tail proteins (gp11 and gp12) and the receptor recognition protein (gp17) of T7 bacteriophage with their counterparts from Klebsiella phage K11, resulting in a synthetic bacteriophage capable of infecting Klebsiella. This study highlights the potential of *in vitro* phage genome synthesis methods to modify the host range of phages by replacing the entire receptor recognition genes (Ando et al., 2015).

Yosef et al. (2017) developed a platform called GOTraP for bacteriophage engineering. In their approach, a T7 phage lacking tail genes (gp11, gp12, gp17) was utilized to infect *E. coli* hosts containing a plasmid carrying tails sourced from various origins, along with an antibiotic resistance marker. Subsequently, the progeny phages were screened with the desired host, allowing only those phages with functional tails, resulting from homologous recombination, to transduce into the target host cells and confer antibiotic resistance. By sequencing the screened bacteria, Yosef et al. (2017) also established a connection between the observed phenotype, which was DNA transduction, and the required genotype, involving mutations in the tail-coding genes that facilitated this transduction process. This method enabled the selection of phages with enhanced transduction efficiency compared to the wild type, as demonstrated by Yosef et al. (2017). However, it should be noted that increased transduction efficiency does not necessarily imply higher infectivity. It is important to acknowledge that both methods, including the one discussed previously, have a common limitation, which is the requirement for homology at the sequence edges of the receptor recognition genes being replaced to meet the criteria for *in vitro* synthesis or homologous recombination (Yosef et al., 2017).

## 5. Study the interaction between phage and bacteria by high throughput method

**Figure 3:**
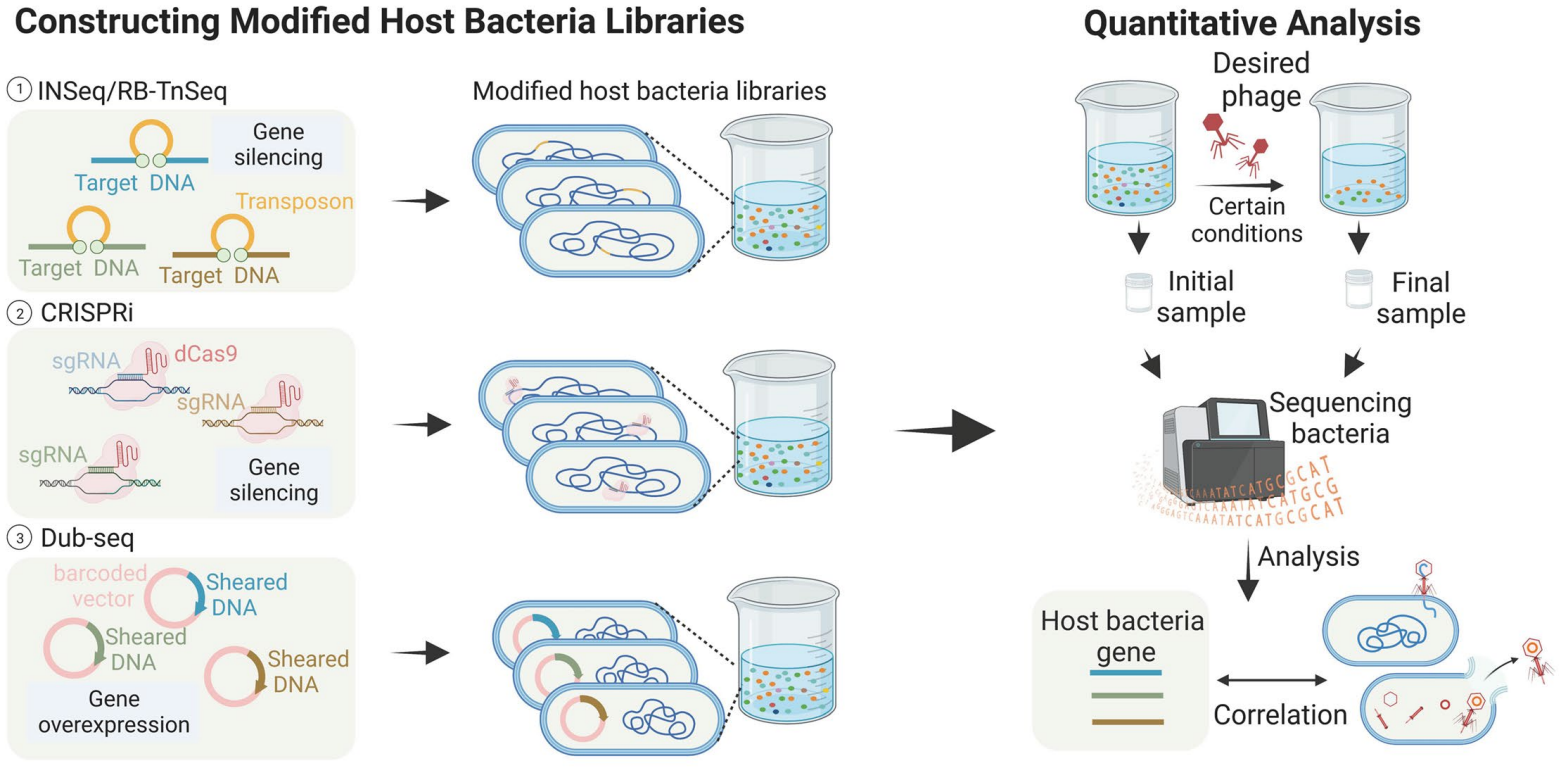
High-throughput analysis of host bacteria genes (images are created with Biorender.com).

**Figure 4:**
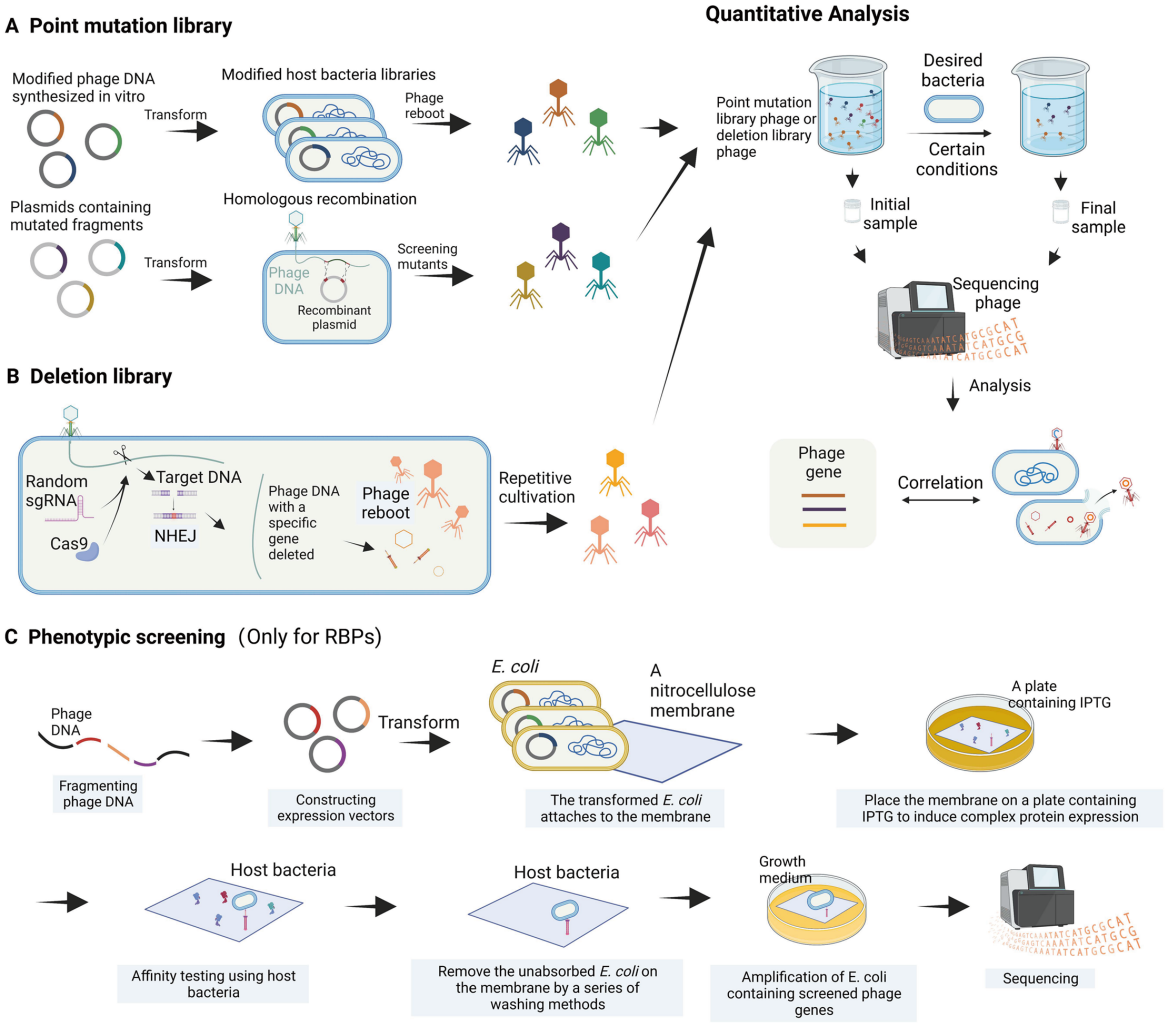
High-throughput analysis of phage genes. **(A)** Construction of a point mutation library. **(B)** Construction of a deletion library. **(C)** Phenotypic screening is employed to identify bacteriophage receptor recognition proteins (images are created with Biorender.com).

The interactions between bacteriophages and their host bacteria are complex and not yet fully understood. However, the development of high-throughput research methods has greatly improved our understanding of these interactions at the molecular level. In this review, we will provide an overview of several approaches, encompassing high-throughput methods for analyzing host bacterial genes (Figure 3) as well as high-throughput methods for analyzing bacteriophage genes (Figure 4).

### 5.1. Transposase-based methods

Transposon insertion sequencing (TIS) has been successfully used to detect genes essential for bacterial growth (Chao et al., 2016; Peters et al., 2016; Liu et al., 2017). This class of genome-wide loss-of-function (LOF) methods has also been applied to identify genes associated with phage infestation in the host bacteriophage genome. Kortright et al. (2020) explored the utility of Insertion Sequencing (INSeq) for identifying bacterial genes involved in bacteriophage binding. They created a transposon mutant library using a modified transposon, and enriched and differentiated the libraries. The method employed in this study successfully identified bacterial genes associated with receptor binding in T2, T4, T6, and T7 bacteriophages. These genes include outer membrane proteins, such as *OmpA*, *OmpC*, *OmpX*, *LamB*, and *tsx*, LPS-related genes like *waaF*, *waaG*, *gmhB*, and *yaeI*, as well as oxidoreductase proteins like *trxA*. These findings are consistent with previous studies (Yosef et al., 2017; Chen et al., 2020), and additionally demonstrated the successful identification of binding targets in five new bacteriophages, thereby proving the generalizability of the method (Kortright et al., 2020). The RB-TnSeq method, which is similar to INSeq, is also reported (Mutalik et al., 2020). These transposon insertion techniques greatly aid in identifying the receptors contained within bacteria, especially in the studies of new bacteriophages and new bacteria. For example, Mutalik et al. (2020) screened a transposon mutant library of the marine pathogenic bacterium *Vibrio parahaemolyticus* and discovered that the vp0980 mutant (encoding a predicted transmembrane protein) was not infected by bacteriophage OWB. Subsequent experiments confirmed that the binding of bacteriophage tail tube proteins A and B with Vp0980 mediated bacteriophage attachment and subsequent bacterial lysis.

### 5.2. CRISPR-based methods

Compared to transposon insertion methods, CRISPR-based methods do not directly modify DNA, making them more flexible in terms of operation. CRISPRi allows partial silencing of gene function through transcriptional repression to achieve LOF. Rousset et al. (2018) reported a CRISPR-dCas9 screening that utilized a library of approximately 17,220 sgRNAs targeting randomly assigned positions in the *E. coli* chromosome. In this experiment, bacteriophages dissolve bacteria unless they carry sgRNAs that allow them to resist infection. The results showed that phages λ, T4, and 186 employ different host genes when killing *E. coli*, and that three distinct host pathways may be involved in these bacteriophage infections. The advantages of CRISPRi technology over INSeq include the ability to examine the function of host-essential genes during phage infection. Unlike randomly inserted transposons, CRISPRi libraries can be designed in a targeted manner to focus on specific gene sites or subsets. Furthermore, CRISPRi allows for the attainment of moderate inhibition levels by targeting gene template strands or utilizing sgRNAs with varying levels of mismatches (Rousset et al., 2018).

### 5.3. Gain-of-function genetic techniques

Gain-of-function (GOF) genetic techniques have been utilized in phage-host interaction studies, where gene overexpression can effectively reveal dominant negative mutations, antisense RNAs, or other genes involved in phage resistance. Mutalik et al. (2020) employed dual-barcoded shotgun expression library sequencing (Dub-seq) to facilitate the overexpression of randomly selected genes in a model *E. coli* system and used this approach to screen and sequence 14 phages. The results showed a degree of similarity in the host genes identified by the two loss-of-function methods (Mutalik et al., 2020). However, Dub-seq diverged from these methods by identifying numerous multicopy repressor genes that encoded diverse functions, shedding light on how changes in host gene expression impact phage resistance differently. This study thus demonstrates the potential of Dub-seq as a useful tool in identifying genes involved in phage resistance.

### 5.4. High-throughput analysis of phage genes

The genomic modification of phages is often more complex than that of host bacteria, and it is challenging to use high-throughput methods to study the function of phage genes when they infect bacteria. Recently, a systematic review article introduced various high-throughput methods for investigating the relationship between bacteriophage sequences and their functions (Huss et al., 2023). The main concept of these methods involves constructing bacteriophage libraries, such as point mutation libraries and deletion libraries, through different bacteriophage engineering techniques, including gene editing using recombination and *in vitro* phage DNA synthesis. These libraries are then screened under different environmental and host conditions (Figure 4). Variants with essential genes for bacteriophage proliferation altered and resulting in functional deficiencies are depleted, while those with beneficial mutations are enriched. The depleted or enriched variants are identified using high-throughput sequencing methods (Figure 4). While this method is theoretically feasible, there have been limited reports on its application thus far. A partially analogous case is the study of T3 bacteriophage receptor-binding protein (RBP) gene recombination and screening (Yehl et al., 2019). In this study, recombination plasmids were constructed to contain the T3 tail fiber (TF) gene, where codons in the loops were substituted with NNK codons through site-directed mutagenesis. By homologous recombination with the bacteriophage genome within the host bacterium, a bacteriophage library with completely random sequences of the TF (loops) was generated (Figure 4A). Yehl et al. (2019) successfully screened and enriched mutant phages capable of infecting T3-resistant *E. coli* by repeatedly co-culturing the library with the host bacterium. Through high-throughput methods, this study provided evidence of the correlation between loop motifs in the T3 phage TF and its infectivity. For the construction of deletion libraries, the CRISPR-Cas system can be utilized. Yuan et al. developed a genome-scale top-down strategy involving the use of the CRISPR-Cas9 system in the host bacterium to randomly target cleavage and subsequently perform non-homologous end joining (NHEJ) of phage DNA, resulting in the random deletion of sequence fragments (Yuan et al., 2022). The progeny phage DNA obtained by repeating these steps several times showed significant simplification compared to the wild-type. This genome-scale top-down strategy, developed by Yuan et al., enables the deletion of non-essential genes in the phage genome. Moreover, by utilizing a random sgRNA library under appropriate conditions, this method can also be employed for the construction of bacteriophage deletion libraries (Figure 4B).

Additionally, Simpson et al. (2016) proposed a high-throughput phenotypic screening approach for RBP genes that entails fragmenting phage DNA, ligating it to an expression vector, transforming it into *E. coli*, and overexpressing the recombinant protein on a nitrocellulose membrane. The binding capability of the nitrocellulose membrane to the phage host bacterium is then assessed, allowing for the identification of phage proteins with high affinity for the host bacterium (Simpson et al., 2016). The *in vitro* phenotypic screening approach (Figure 4C) can help us find the genes of RBPs that interact with the host bacterium, but cannot detect the relevant genes involved in physiological processes.

## 6. Conclusion and future prospects

**Figure 5:**
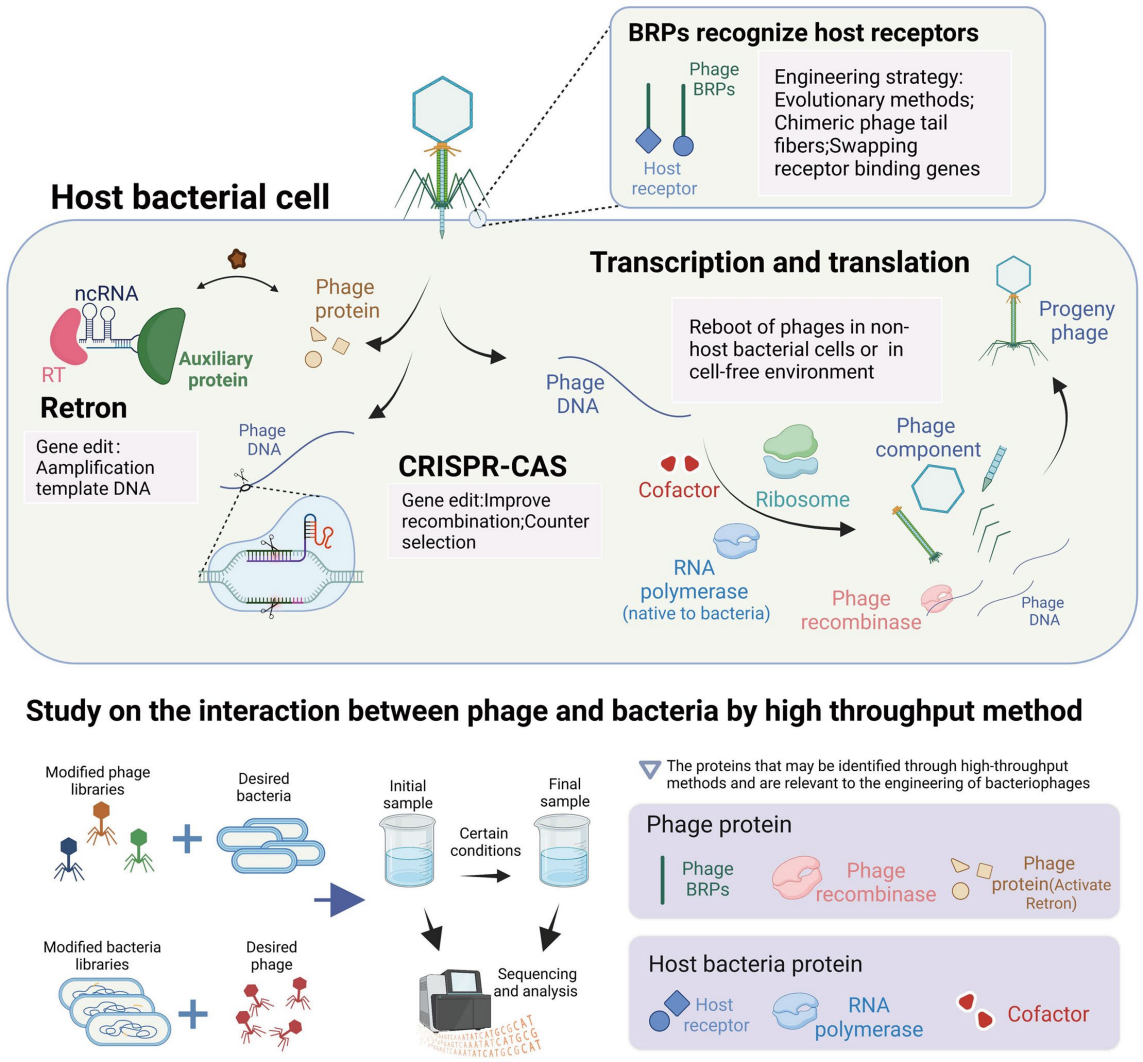
Interaction between bacteriophages and their host and its application in the engineering of bacteriophages. The highlighted content in light pink represents the utilization of interaction mechanisms in the context of phage engineering applications. The content highlighted with a light purple background represents the proteins that can be identified using high-throughput methods and are relevant to the engineering of bacteriophages. These proteins play a crucial role in understanding phage-host interactions and can be targeted for modification and optimization in phage engineering processes (images are created with Biorender.com).

In this review, we summarized the methods and applications of phage engineering that are based on the interaction between phages and their hosts. To further elucidate the connections between these mechanisms and methods, we have presented a conceptual diagram in Figure 5. The figure illustrates various mechanisms of interaction between phages and hosts, along with high-throughput methods for studying phage-host interactions. Additionally, it highlights the applications of engineered phages related to these interactions in light pink background boxes, and identifies relevant proteins that may be identified by high-throughput methods in light purple background boxes.

The tools used for bacteriophage genome editing are derived from the bacterial anti-bacteriophage defense system within bacteria. These tools have been transferred and transformed to function in different bacteria, such as the CRISPR and retron systems. The CRISPR system is used during the editing process to induce double-stranded breaks in bacteriophage DNA and for screening engineered bacteriophages. In contrast, the retron system facilitates the amplification of template DNA in bacterial cells and promotes homologous recombination. Additionally, the lambda red recombinases system, which enhances recombination efficiency, was discovered in bacteriophage genes and has been developed. The reboot of phages outside the host bacteria requires the expression of phage genes. In the non-host bacterial environment, the reboot of phages requires the provision of corresponding enzymes by the bacteria to meet the transcriptional demands of the phages. In the cell-free environment, the reboot of phages directly utilizes the gene expression system from the bacterial cells. The expression of phage genes and the participation of host proteins in the development of cell-free extract (CFE) technology can be revealed, facilitating the development of CFE technology. A crucial goal of phage engineering is the remodeling of phage RNA-binding proteins (RBPs) as it can alter the host range of the phages to meet their therapeutic or other uses. The remodeling approach of RBPs mimics the evolution system of phages under bacterial defense pressure. For example, the introduction of artificial RBP mutations can allow the phages to retarget bacteria indicating receptor mutations, while the replacement or integration engineering of RBPs is similar to the gene exchange among phages in nature, allowing for a significant change in the host range of the phages. In conclusion, the interaction between phages and bacteria provides us with tools for phage engineering and guidance for their development. High-throughput methods offer valuable insights into the proteins involved in phage-host interactions and their application in phage engineering. These methods are instrumental in identifying phage receptor recognition proteins and bacterial receptors, essential for modifying phage receptor range and restoring infectivity against resistant host bacteria. Additionally, high-throughput methods can be used to discover phage recombinases, enzymes involved in phage DNA replication (Murphy, 2012), enabling enhanced recombination processes in phage gene engineering, such as lambda red recombinases (Fehér et al., 2012). Furthermore, the identification of phage proteins that activate the retron system can facilitate the engineering of phages with retron resistance. In terms of host bacteria, high-throughput methods assist in identifying RNA polymerases and cofactors involved in phage DNA transcription and assembly, crucial factors affecting the efficiency of *in vitro* phage rebooting (Yim et al., 2019; Silverman et al., 2020).

Retrons are a current topic of research that holds great promise due to their small size and immunological specificity. If we can fully understand the mechanism by which retrons specifically recognize bacteriophages, they may replace the CRISPR-Cas system for negative selection in bacteriophage engineering. Furthermore, when combined with reverse transcription editing templates, retrons could improve the efficiency of homologous recombination and offer a promising method for bacteriophage gene editing within the host bacterium. Besides, the development of cell-free expression (CFE) technology can provide us with more knowledge regarding the transcriptional mechanisms of bacteriophages. This, in turn, can assist us in synthesizing a greater number of engineered bacteriophages outside of host cells and reduce the reliance on engineering methods within host cells, which are limited by their lower transformation efficiency. CFE technology holds high prospects for application due to its efficiency and safety. In the process of CFE, the production of phages does not generate endotoxins produced by lysed bacteria, simplifying the purification process and reducing culturing time. As a result, the rapid development of CFE technology can effectively meet the demands of phage therapy. Furthermore, high-throughput methods for studying the interaction between phages and host bacteria are efficient and can help us identify genes that play a role in their interaction. This knowledge can effectively guide our modification of phages, leading to more efficient and effective phage therapy.

## Author contributions

H-JJ: data curation, writing original draft, and software analysis. P-PJ, SY, L-KB, and GY: data curation and writing editing. D-SP: conceptualization, funding acquisition, project administration and writing editing. All authors contributed to the article and approved the submitted version.

## Funding

This work was supported by the Chongqing Medical University Talent Project (Nos. R4014 to D-SP, and R4020 to P-PJ), National Natural Science Foundation (NSFC) of China (No. 32200386 to P-PJ), and China-Sri Lanka Joint Research and Demonstration Center for Water Technology, China-Sri Lanka Joint Center for Education and Research, Chinese Academy of Sciences, China.

## Conflict of interest

The authors declare that the research was conducted in the absence of any commercial or financial relationships that could be construed as a potential conflict of interest.

## Publisher’s note

All claims expressed in this article are solely those of the authors and do not necessarily represent those of their affiliated organizations, or those of the publisher, the editors and the reviewers. Any product that may be evaluated in this article, or claim that may be made by its manufacturer, is not guaranteed or endorsed by the publisher.
